# Endoplasmic Reticulum Calcium Pumps and Cancer Cell Differentiation

**DOI:** 10.3390/biom2010165

**Published:** 2012-03-05

**Authors:** Béla Papp, Jean-Philippe Brouland, Atousa Arbabian, Pascal Gélébart, Tünde Kovács, Régis Bobe, Jocelyne Enouf, Nadine Varin-Blank, Ágota Apáti

**Affiliations:** 1 Institut National de la Santé et de la Recherche Médicale, Inserm UMR U978, UFR SMBH Université Paris 13-Paris Nord, 74, rue Marcel Cachin 93000 Bobigny, France; 2 Service d’Anatomie et Cytologie Pathologique, Hôpital Lariboisière, 1, rue Ambroise Paré, 75010 Paris, France; Email: jean-philippe.brouland@lrb.aphp.fr; 3 Inserm UMR U 940, IUH Université Paris 7-Paris Diderot, 16, rue de la Grange aux Belles, 75010 Paris, France; Email: atoussa-arbabian@hotmail.fr; 4 Department of Laboratory Medicine and Pathology, Cross Cancer Institute and University of Alberta, 11560 University Avenue, Edmonton, AB T6G 1Z2, Canada; Email: pascalgelebart@hotmail.com; 5 Semmelweis University, Department of Medical Biochemistry, Tűzoltó u. 37-47, H-1094-Budapest, Hungary; Email: kovax@kkk.org.hu; 6 Inserm UMR U770, Université Paris-Sud 11. 80, rue du Général Leclerc, 94276 Le Kremlin-Bicêtre, France; Email: regis.bobe@inserm.fr; 7 Inserm UMR U689, Université Paris 7-Paris Diderot, Hôpital Lariboisière, 1, rue Ambroise Paré, 75010 Paris, France; Email: jocelyne.enouf@inserm.fr; 8 Institut National de la Santé et de la Recherche Médicale, Inserm UMR U978, UFR SMBH Université Paris 13-Paris Nord, 74, rue Marcel Cachin 93000 Bobigny, France; Email: nadine.varin@inserm.fr; 9 Membrane Research Group of the Hungarian Academy of Sciences, Diószegi út 64, H-1113-Budapest, Hungary; Email: apati@biomembrane.hu

**Keywords:** calcium signalling, endoplasmic reticulum, SERCA, cancer, cell differentiation

## Abstract

The endoplasmic reticulum (ER) is a major intracellular calcium storage pool and a multifunctional organelle that accomplishes several calcium-dependent functions involved in many homeostatic and signaling mechanisms. Calcium is accumulated in the ER by Sarco/Endoplasmic Reticulum Calcium ATPase (SERCA)-type calcium pumps. SERCA activity can determine ER calcium content available for intra-ER functions and for calcium release into the cytosol, and can shape the spatiotemporal characteristics of calcium signals. SERCA function therefore constitutes an important nodal point in the regulation of cellular calcium homeostasis and signaling, and can exert important effects on cell growth, differentiation and survival. In several cell types such as cells of hematopoietic origin, mammary, gastric and colonic epithelium, SERCA2 and SERCA3-type calcium pumps are simultaneously expressed, and SERCA3 expression levels undergo significant changes during cell differentiation, activation or immortalization. In addition, SERCA3 expression is decreased or lost in several tumor types when compared to the corresponding normal tissue. These observations indicate that ER calcium homeostasis is remodeled during cell differentiation, and may present defects due to decreased SERCA3 expression in tumors. Modulation of the state of differentiation of the ER reflected by SERCA3 expression constitutes an interesting new aspect of cell differentiation and tumor biology.

## 1. ER Calcium Sequestration: An Essential Component and Key Modulator of Cell Activation and Survival

Calcium is actively accumulated into the endoplasmic reticulum (ER) from the cytosol by Sarco/Endoplasmic Reticulum Calcium ATPase (SERCA)-type calcium pumps. By using the energy of ATP hydrolysis, these enzymes, located in the ER membrane, generate a strong calcium ion concentration gradient between the ER lumen (high micromolar [1,2]) and the cytosol (low nanomolar [3]). ER calcium storage is essential for the initiation of calcium-dependent cell activation. The activation of many normal plasma membrane receptors (EGF, FGF, PDGF, chemokine, bioactive peptide receptors, *etc*.), but also oncogenic mutant receptor activity [4,5] leads, in parallel with the activation of other signaling pathways, to the activation of phospholipase C enzymes and the hydrolytic cleavage of membrane phosphatidylinositol-4,5-*bis*phosphate into diacylglycerol and inositol-1,4,5-*tris*phosphate (IP3) [3]. Binding of IP3 to IP3-receptor calcium channels (IP3R) leads to IP3R opening and to calcium release from the ER into the cytosol. ER calcium depletion upon IP3R opening then leads to calcium influx into the cytosol from the extracellular space through Orai-type plasma membrane calcium channels [6], and TRP-type channels, as well as non-capacitative calcium influx can also contribute to calcium entry into the cell. Opening of Orai-type channels is induced by STIM-1, an integral ER membrane protein which is in turn activated by the dissociation of calcium from its ER-luminal calcium binding region when ER calcium decreases during IP3-induced calcium release [7,8]. Calcium release from the ER combined with capacitative calcium influx from the extracellular space leads to markedly increased cytosolic calcium levels and the activation of key calcium-dependent enzymes such as protein kinase-C isoforms, calcineurin, calpains, calmodulin dependent kinases and other calmodulin binding proteins involved in cell activation [9,10].

The amplitude of calcium release from the ER depends on the magnitude of the calcium concentration gradient between the ER and the cytosol. Moreover, the variations of intra-ER calcium levels are known to modulate the opening of IP3R calcium channels by IP3 [3,11], and are essential for STIM activation [12,13]. Therefore, the precise regulation of the intra-ER calcium concentration constitutes an important mechanism to adjust the sensitivity of a cell to calcium mobilizing stimuli. Because calcium is accumulated in the ER exclusively by SERCA enzymes, SERCA-dependent calcium transport constitutes a key nodal point in the control of cell activation. In addition, because during cell activation SERCA enzymes rapidly re-accumulate part of the calcium released into the cytosol, SERCA activity exerts an important influence on the amplitude, the shape, as well as the frequency of cellular calcium peaks and oscillations [14,15,16,17,18,19,20] and therefore on cell activation [21,22].

In addition, calcium accumulated by SERCA enzymes is required also for intra-ER functions such as chaperoning of newly synthesized proteins transiting through the organelle [23,24,25,26]. Several ER resident chaperones such as calreticulin or calnexin bind and require calcium for activity [23,25,27]. Therefore, defects of ER luminal calcium homeostasis can lead to defects of protein maturation and to the accumulation of misfolded proteins in the ER, that activates various adaptive ER stress responses or, if overwhelming, leads to cell death [28,29,30,31,32].

Availability of calcium ions in the ER lumen for (1) second-messenger-induced calcium release; (2) the control of capacitative calcium influx; and (3) intra-ER chaperone activities are all critically dependent on proper SERCA function. SERCA-type enzymes occupy therefore a critical position in cellular calcium homeostasis and signaling, and subtle changes in SERCA expression and activity can have far-reaching consequences for the dynamics of calcium signaling and the behavior or the survival of the cell. 

The position that SERCA-dependent calcium transport occupies in cell signaling makes it also a promising pharmacological target. For example, SERCA inhibition for the targeted therapy of prostate cancer is currently being evaluated using peptide conjugates of thapsigargin, a highly potent and selective SERCA inhibitor. The peptide conjugates are hydrophilic and therefore remain extracellular and thus inactive when administered intravenously. However, hydrolysis of the conjugate by PSA, a prostate-specific peptidase will lead to the release of free thapsigargin in the vicinity of the cells, its diffusion into the cell, SERCA inhibition and induction of cell death [33,34].

## 2. The SERCA Multigene Family, Co-Expression of SERCA2 and SERCA3 Proteins

Three SERCA genes are known in the human genome (ATP2A1, ATPA2 and ATP2A3), that by alternative splicing can generate several protein isoforms that differ in their C-terminal regions [35,36,37,38,39]. The expression of SERCA isoenzymes is tissue dependent and developmentally regulated. Whereas SERCA1a and 1b are expressed in adult and neonatal fast twitch skeletal muscle, respectively, SERCA2a is expressed in cardiomyocytes, and SERCA2b is abundant in smooth muscle cells. A minor isoform, SERCA2c has also been detected in various tissues [40]. Although abundantly expressed in smooth muscle, SERCA2b has also been detected in almost all non-muscle cell types as well, indicating that SERCA2b is a ubiquitous isoform involved in calcium uptake in the ER in most cells. The third member of the SERCA family, SERCA3 bears approximately 80% homology with other SERCA isoforms [37,41]. The ATP2A3 gene can give rise to six known SERCA3 isoforms that arise by alternative splicing in the 3’ region of the transcripts [39,42,43]. Comparative analysis of the localization and of the biochemical characteristics of various SERCA isoforms revealed significant differences. Transport activity is stimulated by calcium in a concentration-dependent manner [44,45,46]. When the calcium concentration dependency of calcium transport by various SERCA isoforms was compared, it has been shown that the apparent calcium affinity (K_Ca_^2+^, as defined by the calcium concentration that leads to half-maximal induction of transport) of all SERCA3 isoforms is weaker (approximately 1.2 μM) than that of other isoforms, and in particular of SERCA2b (0.2 μM) [35,47,48,49,50,51]. Based on this observation SERCA2b is thought to be a more “stringent” calcium pump than SERCA3: whereas SERCA2b-dependent calcium sequestration is fully active already above the 0.2 μM cytosolic calcium concentration range, fully active calcium sequestration by SERCA3 would be observed only above 1.2 μM calcium, and SERCA3 would pump calcium very weakly at around 0.2 μM [35,37,39]. 

A new level of complexity has been discovered when it was shown that in several cell types SERCA2b and SERCA3 enzymes are expressed simultaneously [52,53]. In cells of hematopoietic origin (lymphoid, myeloid, megakaryocytic cells, cell lines, as well as platelets), insulin-secreting pancreatic β-cells, gastric and colonic epithelium, as well as Purkinje neurons, SERCA2 and SERCA3 enzymes can be found in various amounts simultaneously. SERCA3 is expressed also in vascular endothelial cells, and expression levels vary according to the proliferative state and the anatomic location of the cells [54]. Several excellent reviews are available about SERCA structure [42,50,55,56,57,58,59], function [35,37,38,39,57,60,61], knock-out animal models [62,63] and genetic diseases [50,64,65,66,67], as well as about the role of calcium signaling in cancer [68,69]. With the aim of attracting attention to the remodeling of ER calcium homeostasis in cancer, we will briefly summarize here available data on the modulation of the expression of SERCA enzymes in several *in vitro* models of cancer cell differentiation, and on the patterns of SERCA3 protein expression in various human tumors and corresponding normal tissue *in situ*.

### 2.1. Myeloid Leukemia

Acute promyelocytic leukemia (APL) is a myeloid malignancy in which cells blocked at the promyelocyte stage of myeloid differentiation accumulate. In most cases leukemic cells carry the t(15;17)(q24;q21) chromosomal translocation that leads to the expression of the PML/RARα (Promyelocytic Leukemia/Retinoic Acid Receptor-alpha) fusion oncoprotein. At physiological (nanomolar) all-*trans*-retinoic acid concentrations PML/RARα acts as a dominant negative inhibitor of gene expression that, by binding to target gene promoters and the recruitment of nuclear co-repressors inhibits granulocytic differentiation. At higher, pharmacologic concentrations (near micromolar), binding of all-*trans* retinoic acid to PML/RARα relieves this inhibition, leading to the dissociation of co-repressors, the recruitment of transcriptional co-activators and expression of target genes, followed by the proteolytic degradation of the PML/RARα oncoprotein [70,71,72]. Treatment by all-*trans* retinoic acid (ATRA) leads to growth arrest and to the terminal neutrophil granulocytic differentiation of APL cells *in vitro*, as well as *in vivo*, and constitutes the first example of molecularly targeted therapy of leukemia. When combined with cytotoxic treatments aimed at the elimination of the leukemia initiating cells, ATRA is highly successful for the treatment of APL [73]. 

When the neutrophil granulocytic differentiation of cell lines or freshly isolated APL cells is induced by ATRA, significant changes of SERCA expression occur [74]. Similarly to all cell lines of hematopoietic origin tested so far, untreated cells express SERCA2, as well as SERCA3. However, during differentiation SERCA3 expression is selectively induced, whereas that of SERCA2 is decreased, or is not modified significantly [74]. The induction of SERCA3 expression could be observed also during cell differentiation induced by an RARα-specific synthetic agonist, and ATRA-induced differentiation, as well as SERCA3 induction was inhibited by an RARα-selective antagonist [74]. Importantly, SERCA3 expression was induced during the differentiation of the cells induced by cAMP analogues as well, and SERCA3 expression was not modified by ATRA in cells refractory to the differentiation-inducing effect of the drug [74]. Taken together, these observations show that the induction of SERCA3 expression is an integral part of the neutrophil granulocytic differentiation program of APL cells. 

The functional consequences of the modulation of SERCA expression on calcium transport activity were investigated in HL-60 cells. ATRA-induced neutrophil granulocytic differentiation of HL-60 cells leads to increased SERCA3 expression, whereas SERCA2 expression is at the same time decreased [74]. When ATP-dependent ^45^Ca^2+^ accumulation into microsomal membrane preparations obtained from control and differentiated HL-60 cells was compared, calcium accumulation into the SERCA3-associated compartment was markedly increased, whereas calcium accumulation into the SERCA2-associated pool was decreased [74]. This indicates that the modulation of SERCA expression leads to the functional remodeling of calcium transport and a shift of calcium uptake into a SERCA3-associated storage pool.

Induction of SERCA3 expression could also be observed during the megakaryocytic differentiation of various human erythro-megakaryoblastic leukemia cell lines induced by protein kinase C activating phorbol esters [75]. Platelets, that correspond to the ultimate stage of megakaryocyte differentiation contain very high amount of SERCA3 protein [53,76]. Induction of SERCA3 expression during *in vitro* differentiation of megakaryocytic cell lines, expression of SERCA3 in mature normal human megakaryocytes and circulating platelets indicate that induction of SERCA3 expression is part of the differentiation program of this lineage. 

SERCA2 and SERCA3 signal intensities on Western blots with isoform-specific [52,74], as well as *pan*-SERCA antibodies [53] lay within the same order of magnitude, and ^32^P-labeled phosphoenzyme levels for SERCA2 and SERCA3 are also roughly comparable in platelet membranes [53]. In addition, ^45^Ca^2+^-transport measurements on platelet- as well as HL-60 cell-derived microsomal membrane preparations suggest that SERCA2 and SERCA3 contribute to ER calcium uptake to comparable extents [74,77]. These observations indicate that the contribution of SERCA2 and SERCA3 to total SERCA function lies within the same order of magnitude. In other words, SERCA3 is not a minor or marginally expressed isoform when compared to SERCA2 in differentiated cells.

### 2.2. Colon Carcinoma

Normal colonic epithelium is a rapidly renewing tissue in which asymmetric division of epithelial stem cells located in the region of the crypt base is followed by the proliferation and the differentiation of upward migrating epithelial cells, which thereafter undergo apoptosis in the surface epithelium [78]. Tumorigenesis in the colonic epithelium is regarded as a multistep process whereby the accumulation of mutations that inactivate tumor suppressor genes and activate oncogenes leads to the stepwise acquisition of neoplastic phenotypes of increasing malignant potential [79,80]. This is best illustrated by the adenoma to adenocarcinoma sequence: mutations in the APC/β-catenin/TCF4 pathway induce the formation of low grade benign tumors (adenomas) that upon the acquisition of further mutations (k-Ras, SMAD-4, p53 and others) increase in grade and then become malignant (adenocarcinomas) [79,81]. Premalignant, as well as malignant lesions in the colon can be graded according to histological differentiation, and the low grade to high grade adenoma to *in situ* and invasive well/moderately/poorly differentiated adenocarcinoma sequence corresponds to the sequential loss of phenotypic differentiation and increased malignant potential. Small lesions called hyperplastic polyps of Morson, which, in contrast to adenomas, are devoid of significant potential to develop into carcinoma can also arise in the colon [82,83,84].

When SERCA3 expression is investigated in the colon by immunohistochemistry, strong SERCA3 expression can be observed in the epithelial cells, and staining increases from the region of the crypt base where colonic epithelial stem cells are located towards the surface epithelium [85]. This indicates that SERCA3 is abundantly expressed in the differentiating normal colonic epithelium, and a similar SERCA3 staining pattern can be observed also in hyperplastic polyps. On the other hand, SERCA3 expression is progressively decreased along the adenoma/adenocarcinoma sequence: in contrast to normal epithelium that strongly expresses SERCA3, staining is globally decreased and heterogeneous in adenomas, with a more marked decrease observed in high grade lesions, is very low in well differentiated adenocarcinomas, and barely detectable or absent in moderately and poorly differentiated carcinomas.

Colon carcinoma cell lines can be induced to undergo differentiation *in vitro* by treatment with short chain fatty acid-type histone deacetylase inhibitors such as butyrate or valerate, butyrate releasing prodrugs or ω-aryl-substituted short chain fatty acid analogues such as phenylbutyrate [86]. Short chain fatty acid-induced differentiation is physiologically relevant. Short chain fatty acids present in the colon lumen due to the fermentation of dietary fibers by the colonic flora are thought to induce differentiation of the normal epithelium and of microscopic precancerous lesions thereby contributing to the cancer-preventive effects of dietary fiber consumption [87,88]. In addition, the Caco-2 colon adenocarcinoma cell line spontaneously undergoes differentiation when cultured in post-confluent conditions. This can be detected by morphological (formation of a polarised epithelial monolayer with brush border membrane and tight junctions), functional (transcellular solute transport, transepithelial electric resistance), as well as biochemical criteria (induction of the expression of markers such as dipeptidyl peptidase 4, carcinoembryonic antigen, sucrase-isomaltase or the isoform switch of the ZO-1 tight junction protein) [89]. Induction of colon and gastric carcinoma cell lines by various differentiation-inducing treatments including short chain fatty acids and analogues, as well as the spontaneous differentiation of Caco-2 cells is associated with the selective induction of the expression of SERCA3 protein [86]. In addition, the inhibition of the APC/β-catenin/TCF4 pathway in colon cancer cells by the forced expression of a transfected, dominant negative TCF4 variant also leads to increased SERCA3 expression [85].

The effect of butyrate treatment on cellular calcium homeostasis was investigated in the Kato-III gastric carcinoma cell line, in which treatment leads to a marked induction of SERCA3 expression, whereas SERCA2 levels are at the same time decreased. As shown by Fura-2 calcium fluorimetry, butyrate treatment is associated with increased resting cytosolic calcium levels and decreased thapsigargin-sensitive intra-ER calcium storage [86].

Taken together, these observations indicate that SERCA3 expression is lost during the multi-step process of colon carcinogenesis, that decreased SERCA3 expression is an early marker of colon tumorigenesis, and that SERCA3 expression is induced during colon and gastric cancer cell differentiation, a process during which the calcium homeostasis of the cell is modified.

### 2.3. Breast Cancer

Breast tumorigenesis is a rather complex process in which several parallel molecular oncogenic mechanisms operate in a somewhat combinatorial manner [90,91,92,93]. This leads to the formation of several types of preneoplastic lesions with various types and degrees of dysplasia, and of various cancer types such as ductal and lobular carcinoma [94]. Most breast carcinomas are thought to arise in the terminal ductal lobular units that consist of acinar secretory cells, myoepithelial cells and the cells of the intralobular duct [94]. The classification of breast neoplasia can be performed based on histological, immunophenotypic and hormonal criteria, as well as by the detection of genetic mutations associated with different tumor types. Classification is pertinent for prognosis and response to various types of treatment [92]. In addition, whole genome and transcriptome analyses are currently used for the molecular classification of breast cancer [90,93,95]. However, because of the interconnectedness of several, not sufficiently known oncogenic mechanisms [92], different classification methods are not always concordant, and the behaviour and the response to treatment of individual tumors assigned to the same currently used categories may differ significantly.

**Figure 1:**
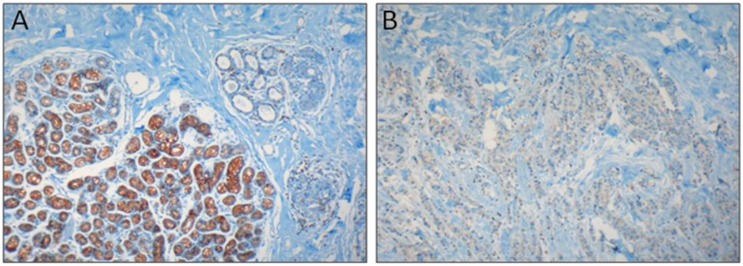
SERCA3 expression in normal breast acini and in invasive lobular breast carcinoma. SERCA3 expression was detected by immunohistochemistry with the avidin-biotin-peroxydase method and 3,3’-diaminobenzidine chromogenic substrate. In normal breast (**A**) strong SERCA3 staining (brown) is observed in the acinar cells of lobules (lower left), and staining of normal ducts is weaker (upper right). When compared to normal acini, SERCA3 expression is markedly decreased in invasive lobular carcinoma (**B**). Tissue was counterstained with hematoxylin (blue).

In order to investigate the role of endoplasmic reticulum calcium biology in breast tumorigenesis, SERCA3 expression was investigated by immunohistochemistry in normal breast, in various preneoplastic lesions and in invasive ductal and lobular breast carcinoma [96]. Whereas normal breast acinar epithelial cells displayed a strong SERCA3 staining, SERCA3 expression was markedly decreased already in very early benign lesions such as adenosis and lobular hyperplasia without atypia, and remained low in lobular carcinoma. This indicates that SERCA3 expression becomes anomalous already at the earliest morphologically detectable stages of lobular dysplasia and remains thereafter low at further stages of lobular tumorigenesis [96] (Figure 1).

In invasive ductal carcinomas SERCA3 expression was globally decreased when compared to normal ducts, and, although variable, was inversely correlated with the Elston-Ellis grade, with the loss of steroid hormone receptor expression, as well as with triple negative (estrogen-, progesterone-receptor and HER-2 negative) status. These observations, combined with the analysis of tumor groups stratified simultaneously for markers such as hormone receptor expression and proliferative index or nuclear grade, showed that SERCA3 expression is inversely correlated with tumor differentiation and the degree of aggressiveness/malignancy of ductal carcinoma of the breast [96].

### 2.4. T Lymphocyte Activation

Calcium signaling plays an important role in T cell activation. Activation of the T-cell receptor complex leads to the hydrolysis of membrane phosphatidyl-inositol-4,5-*bis*phosphate by phospholipase Cγ into diacylglycerol (DAG) and inositol-1,4,5-*tris*phosphate (IP3). This leads to calcium mobilization from the ER, protein kinase C and calcineurin activation, and the activation of NF-κB and NF-AT-type transcription factors that orchestrate T cell activation, the acquisition of a blastic phenotype and lead to intense IL-2-dependent cell proliferation [97].

The effects of IP3 and DAG can be mimicked, respectively, by a calcium ionophore (ionomycin) and a phorbol ester (PMA) *in vitro*. Treatment of the Jurkat (clone E6-1) human T cell line, a widely used model of T lymphocyte activation, by PMA and ionomycin leads to cell activation, as detected by the induction of the expression of the α chain of the IL-2 receptor and IL-2 secretion. When Jurkat E6-1 cells are treated with PMA plus ionomycin, cell activation is accompanied by a strong and selective down-regulation of SERCA3 expression, whereas SERCA2 levels are, at the same time, slightly increased [98]. Interestingly, SERCA3 down-regulation, as well as IL-2 secretion could be induced only by a combined treatment by PMA and ionomycin, whereas treatments by either drug alone were without effect [98]. In addition, T cell activation, as well as the down-regulation of SERCA3 could be inhibited by cyclosporine-A or FK-506 (tacrolimus) [98], clinically used immunosuppressive drugs [99] that by inhibiting calcineurin-induced NF-AT dephosphorylation block T cell activation.

### 2.5. B Lymphocyte Immortalization

Epstein-Barr virus (EBV), a human gammaherpesvirus can immortalize human B lymphocytes by establishing a state of latent infection in which the virus is transmitted during mitosis to daughter cells in an episomal form [100,101]. During immortalization, resting B lymphocytes acquire a proliferating, activated lymphoblastic phenotype induced by the expression of a limited set of viral genes including EBNA2 (Epstein-Barr virus nuclear antigen-2) and LMP-1 (latent membrane protein-1). EBV-induced immortalization is involved in the formation of several lymphoid malignancies including a large fraction of Burkitt’s lymphomas, Hodgkin lymphoma, T/NK lymphomas, lymphomas of immunocompromised individuals, as well as of nasopharyngeal carcinoma and a subset of gastric carcinoma [102,103]. EBNA2, a major viral transactivator and activator of the Notch transcriptional regulatory pathway modulates the expression of several cellular, as well as viral genes including LMP-1. Expression of LMP-1 (considered as a truncated, constitutively active viral analogue of receptors belonging to the TNFα receptor family, that functionally resembles CD40, a key receptor in normal B cell activation) leads to the activation of several signaling pathways leading to NF-κB, AP-1, MAPK and Akt activation [104,105,106,107,108]. The reprogramming of signaling pathways involved in the control of survival and of the state of activation of resting B cells by EBV infection leads to the emergence of autonomously proliferating immortalized lymphoblastoid cell lines.

The investigation of EBV-related effects on the cellular level is greatly facilitated by the availability of pairs of EBV-negative and corresponding latently EBV-infected cell lines. EBV-negative Burkitt’s lymphoma cell lines were infected by EBV *in vitro*, and latently infected cell lines were established [109,110]. Compared to the parental EBV-negative cells, latent EBV infection of the cells leads to significantly decreased SERCA3 expression, whereas SERCA2 levels are at the same time increased. Importantly, the modulation of SERCA expression by EBV was observed only in cell lines infected with a fully immortalizing EBV strain (B95.8 virus), whereas infection with the non-immortalizing P3HR-1 virus strain (in which LMP-1 expression is deficient due to a deletion in the EBNA2 sequence and consequent loss of trans-activation of LMP-1 expression by EBNA2 [111]), SERCA expression was not modified [112]. Investigation of the effect of individual viral proteins on SERCA expression using inducible expression vectors stably transfected into EBV-negative cells has shown, that whereas EBNA2 expression was without effect, SERCA3 expression was selectively down-regulated in cells expressing LMP-1 in the absence of any other EBV product [112]. In latently infected cells, as well as upon LMP-1 expression, increased calcium storage in a thapsigargin-sensitive, presumably SERCA2-associated intracellular calcium pool was observed [112].

SERCA3 expression was also investigated by immunohistochemistry in normal lymph nodes. A strong labeling was obtained in the mantle zone of lymphoid follicles where resting B lymphocytes reside, whereas in germinal centers where activated and proliferating centroblast and centrocytes are located, SERCA3 staining was considerably weaker [112]. Observations on the effect of EBV infection and normal B lymphocyte activation taken together indicate that the down-regulation of SERCA3 expression induced by LMP-1 during EBV-induced immortalization mimics a phenomenon taking place during antigen-dependent activation of normal B lymphocytes in germinal centers. In addition, because SERCA3 down-regulation occurs also during the activation of T lymphocytes as shown in Jurkat cells, it is tempting to propose that the selective down-regulation of SERCA3 expression is a general phenomenon involved in lymphocyte activation in the T, as well as the B lineage. 

## 3. Discussion

### 3.1. SERCA3: A New Marker of Cell Differentiation

Several lines of evidence show that SERCA3 expression undergoes significant quantitative modifications when the state of differentiation or activation of various cell types changes. In several independent model systems of differentiation such as retinoic acid-induced differentiation of acute promyelocytic leukemia cells, phorbol ester-induced differentiation of megakaryoblastic cell lines, or short chain fatty acid-induced, as well as spontaneous differentiation of colon carcinoma cells, differentiation, detected by a multitude of established markers, is accompanied by a marked induction of SERCA3 protein expression, whereas the expression of the simultaneously expressed SERCA2 isoenzyme is much less modified, or is in fact often decreased. In addition, fully differentiated normal cells that correspond to the final step of these differentiation programs (such as platelets or normal colonic surface epithelial cells) express SERCA3 abundantly. Moreover, when investigated in neoplastic tissue *in situ*, a loss of SERCA3 expression is observed when compared to the corresponding normal, differentiated cell type, and the loss of SERCA3 expression is proportional to the degree of histologically observable loss of cell differentiation. This phenomenon has been observed when benign, precancerous and malignant lesions were studied comparatively in the colonic epithelium, as well as in breast epithelial lesions of various degrees of dysplasia or malignancy. Importantly, SERCA3 expression has been shown to decrease already at very early steps of dysplasia in colon adenomas, as well as in lobular breast lesions, and remains low, or becomes undetectable at more advanced stages of tumorigenesis and malignant transformation. Down-regulation of SERCA3 expression could also be observed during the acquisition of an activated phenotype during T, as well as B lymphocyte activation and B cell immortalization, processes associated with proliferation and the acquisition of a blastic phenotype.

These observations show that when a cell undergoes phenotypic changes such as differentiation, activation or transformation, intracellular calcium sequestration by SERCA-dependent calcium pumping is modified in several cell types, and SERCA3 expression is a pertinent phenotypic marker of this process.

### 3.2. Remodeling of ER Calcium Homeostasis during Differentiation

The modification of the SERCA2 to SERCA3 molar ratio can have significant functional consequences on ER calcium homeostasis, handling and availability for signaling functions. The calcium concentration dependence of calcium transport (as defined by the apparent calcium affinity of transport, K_Ca_^2+^) of SERCA2b (K_Ca_^2+^ ≈ 0.2 μM) and SERCA3 (K_Ca_^2+^ ≈ 1.2 μM) is distinct. As pointed out earlier [37,39], this calcium concentration range corresponds approximately to the concentration range in which cytosolic calcium levels vary between the resting and activated state. In a very simplified manner this means that whereas SERCA2b-dependent calcium sequestration in the ER is almost fully active already at resting cytosolic calcium levels, SERCA3-dependent calcium sequestration becomes active at cytosolic calcium levels encountered only during calcium-dependent cell activation. Therefore, whereas SERCA2b-dependent calcium accumulation into the ER would be expected to be constitutively active, SERCA3-dependent calcium transport may become important only during increased cytosolic calcium levels observed during activation, and thus SERCA3 would only blunt higher cytosolic calcium peaks, and would become active only at a later phase of the calcium peak. SERCA3 may also be associated with ER regions around which cytosolic calcium levels can reach significantly higher concentrations locally, such as regions in the immediate proximity of open calcium channels [113,114,115]. Interestingly, earlier work on IP3-induced release of calcium accumulated in platelet microsomal vesicle preparations in the presence of the PLIM430 SERCA3-specific inhibitory antibody [116,117] had shown that calcium accumulation into the IP3-mobilizable sub-compartment of platelet intracellular calcium stores is performed preferentially by SERCA3 [77]. The association of SERCA3, a lower affinity calcium pump with an ER sub-compartment involved in second messenger-induced calcium release probably permits the cell to mount larger second-messenger-induced calcium release responses upon calcium release, which otherwise would be blunted by SERCA2b. It can also be hypothesized that by limiting futile release/reuptake cycles, the association of SERCA3 with IP3-sensible calcium pools constitutes an energy-efficient mechanism that permits larger calcium release signals before re-sequestration is initiated. This notion is compatible with the observed association of SERCA3 expression with various differentiated cell phenotypes: one may hypothesize that the association of an IP3-sensitive ER sub-compartment with a less “stringent” calcium uptake mechanism is typical of differentiated cell types that respond to various external stimuli by calcium mobilization, whereas ER calcium homeostasis in undifferentiated cells behaves more autonomously. Interestingly, the working hypothesis of SERCA3 being associated with intracellular calcium pools specialized in signaling is compatible also with the observed down-regulation of SERCA3 expression during lymphocyte activation, a process during which cellular calcium signaling is chronically activated [118]. One may hypothesize that SERCA3 down-regulation in this configuration leads to the constitutive depletion of an IP3-sensitive intracellular calcium pool coupled to a chronically activated state of store-operated calcium influx mechanism and sustained calcium-induced activation.

If the distribution of SERCA2b and SERCA3 is heterogeneous within the ER, this may have other interesting consequences for intra-luminal calcium homeostasis too. It can be hypothesized that the association of high and low calcium affinity SERCA pumps such as SERCA2b and SERCA3, respectively, with distinct sub-compartments of the contiguous ER membrane network, and consequent differential calcium uptake in these sub-compartments may lead to the formation of intra-luminal longitudinal calcium gradients and calcium ion migration, even in a resting cell. Such gradients and vectorial calcium fluxes, for example from a SERCA2b-associated region towards a SERCA3-associated one may contribute to the organization of structurally and functionally distinct intra-ER spaces.

### 3.3. Cross-Talk between SERCA Function and the Control of Differentiation

The modulation of SERCA expression is not a simple passive consequence of cell differentiation. Complete SERCA inhibition induces cell death due to ER stress responses. On the other hand, the partial inhibition of SERCA-dependent calcium sequestration by highly specific inhibitors such as thapsigargin, a sesquiterpene lactone that inhibits various SERCA isoenzymes with high affinity (below nanomolar), cyclopiazonic acid or 2,5-di-*tert*-butyl-1,4-benzohydroquinone has been shown to enhance or potentiate ATRA-induced differentiation of acute promyelocytic leukemia cells [119], and combined treatments with SERCA inhibitors and ATRA have been shown to induce cell differentiation in several cell lines that are resistant to differentiation induction by ATRA alone [119]. In addition, SERCA inhibition enhances the expression of carcinoembryonic antigen (CEA), a differentiation marker, during post-confluent differentiation of Caco-2 colon carcinoma cells [85], confers cytokine independency to TF-1 erythroleukemia cells [120], and induces HIV expression in latently infected T cells [121]. Moreover, chronic SERCA inhibition *in vivo* displays tumor-promoting activity. Although in most of these settings it is not possible to clearly assign the observed effect specifically to a given SERCA isoform, these observations show that SERCA function and mechanisms that control several types of cell activation and differentiation are functionally interconnected, and a cross-talk exists between the control of ER calcium sequestration and the regulation of cell differentiation in several cell types. Changes of ER calcium pumping may therefore exert important effects on cell activation and differentiation.

### 3.4. Cellular Calcium Homeostasis: A Heavily Interconnected System

Cellular calcium homeostasis is maintained by the concerted action of many calcium handling proteins in the cell leading to a steady state with very different calcium levels in various cellular compartments. Calcium pumping and release occur simultaneously in a cell. Therefore, the calcium concentration, as well as its changes are determined by the concerted action of the entire set of the calcium homeostatic “toolkit” (pumps, channels, calcium binding proteins and their regulators) present in the cell [122], and individual components of this toolkit can display partial functional redundancy. Importantly, the activity of calcium pumps and channels is critically regulated by calcium itself. Several negative, as well as positive feedback mechanisms, cumulative effects and delayed regulations have been described in this context that are modulated by calcium [11,123,124,125,126]. An in-depth understanding of the functional involvement of the remodeling of ER calcium sequestration due to the modulation of SERCA expression will require a more profound understanding of the complex interactions of calcium handling proteins and of the dynamic behavior of this signaling matrix. The consequences of the modulation of the expression and activity of various SERCA isoforms will depend on the given cell signaling context into which these are integrated in a cell. Although heterozygous knock-out of the SERCA2 gene leads to squamous tumorigenesis in mice with long incubation times [62], the corresponding human condition, Darier disease [127,128] does not appear to predispose to tumor formation, and SERCA3 knock-out mice don’t display a neoplastic phenotype. On the other hand, SERCA inhibitors such as thapsigargin [129] or 2,5-di-*tert*-butyl-1,4-benzohydroquinone [130] are known tumor promoters *in vivo*, and mutations in SERCA2, as well as SERCA3 sequences have been found in several human tumor types [131,132,133]. These observations, when combined with data on SERCA expression in cancers, indicate that ER calcium homeostasis is involved in the establishment of several types of the malignant phenotype. 

Phenotypic dedifferentiation is a hallmark of cancer, and, as shown in several tumor types, the loss of SERCA3 expression is part of this process. The accumulated data, when taken together, suggest that the loss of SERCA3 expression reflects the loss of a signaling function or ER sub-compartment present in differentiated cells. Observations made using SERCA inhibitors have shown that partial down-regulation of ER calcium sequestration may lead to differentiation, or enhance the differentiation-inducing effect of other stimuli [85,119]. Thus, it may be hypothesized, that the expression of SERCA3, a low calcium-affinity pump isoform constitutes a physiological mechanism, by which the cell, in analogy with pharmacological SERCA inhibition, renders the corresponding intracellular calcium pool poised to release more calcium into the cytosol, induce stronger capacitative calcium influx, and therefore activate calcium-dependent effector mechanisms involved in cell differentiation more efficiently.

The detailed understanding of the mechanisms that connect ER calcium signaling to tumorigenesis requires further work. Computer modeling and systems biology-type approaches applied to experimental data will be undoubtedly very informative in this context [15,16,134,135]. Finally, it is interesting to note that the remodeling of cellular calcium homeostasis by the selective modulation of the expression of specific calcium transporter isoforms may not be limited only to SERCA3 and the ER. Indeed, colon cancer cell differentiation has recently been shown to lead to the selective induction of the expression of the PMCA4b plasma-membrane-type calcium pump isoenzyme as well [136,137]. By transporting calcium ions into the extracellular space through the plasma membrane, PMCA-type calcium pumps decrease cytosolic calcium levels and thus contribute to the control of cell activation. The modulation of PMCA expression during differentiation indicates that the remodeling of cellular calcium homeostasis during differentiation, as well as its defects in cancer may in fact involve an entire, yet unknown set of specific components of the calcium homeostatic toolkit.

## 4. Conclusions

Accumulating evidence shows that the remodeling of ER calcium pump expression is part of the differentiation program of several cell types. Differentiation is associated with the selective induction of the expression of SERCA3, a lower calcium affinity calcium pump, which is more permissive for second-messenger-induced calcium release than the simultaneously expressed SERCA2b isoenzyme. The modulation of the expression of SERCA isoenzymes constitutes a new mechanism to fine tune ER calcium uptake according to cell phenotype, function and signaling requirements, and may be involved in the structural organization of the organelle. SERCA3 expression is selectively decreased or lost in many tumors, and this probably reflects the loss of a calcium-dependent function characteristic of fully differentiated normal cells. SERCA3 loss is proportional with histological atypia, and can be observed already in premalignant lesions, indicating that ER calcium homeostasis becomes abnormal already at early steps of the process of tumorigenesis. Anomalies of the cross-talk between SERCA function and the control of cell differentiation constitutes a previously unknown aspect of tumor biology that is potentially amenable to pharmacologic intervention, for example by targeted delivery of SERCA inhibitors to tumors.
